# Identification of Bone Metastatic and Prognostic Alternative Splicing Signatures in Prostate Adenocarcinoma

**DOI:** 10.1007/s10528-023-10367-z

**Published:** 2023-04-03

**Authors:** Jiwen Zhu, Jiayao Zhang, Peng Hu, Mingxiang Fan, Dianwen Song, Huabin Yin, Penghui Yan, Shuyuan Xian, Zhenyu Li, Juanru Guo, Chunling Long, Runping Xu, Runzhi Huang, Tong Meng, Jie Zhang, Zongqiang Huang

**Affiliations:** 1 Department of Orthopedics, The First Affiliated Hospital of Zhengzhou University, 1 East Jianshe Road, Zhengzhou, 450052 China; 2 Division of Spine, Department of Orthopedics, Tongji Hospital affiliated to Tongji University School of Medicine, Shanghai, 200065 China; 3 Tongji University School of Medicine, Shanghai, 200092 China; 4 School of Mathematical Sciences of Tongji University, Shanghai, 200092 China; 5 Department of Orthopedics, School of Medicine, Shanghai General Hospital, Shanghai Jiaotong University, 100 Haining Road, Shanghai, 200065 China; 6 Shanghai First Maternity and Infant Hospital, Tongji University School of Medicine, Shanghai, 201204 China

**Keywords:** Alternative splicing, Bone metastasis, Prognosis, Prostate adenocarcinoma, Signaling pathway, Splicing factor

## Abstract

As the most common nonepithelial malignancy, prostate adenocarcinoma (PRAD) is the fifth chief cause of cancer mortality in men. Distant metastasis often occurs in advanced PRAD and most patients are dying from it. However, the mechanism of PRAD progression and metastasis is still unclear. It’s widely reported that more than 94% of genes are selectively splicing in humans and many isoforms are particularly related with cancer progression and metastasis. Spliceosome mutations occur in a mutually exclusive manner in breast cancer, and different components of spliceosomes are targets of somatic mutations in different types of breast cancer. Existing evidence strongly supports the key role of alternative splicing in breast cancer biology, and innovative tools are being developed to use splicing events for diagnostic and therapeutic purposes. In order to identify if the PRAD metastasis is associated with alternative splicing events (ASEs), the RNA sequencing data and ASEs data of 500 PRAD patients were retrieved from The Cancer Genome Atlas (TCGA) and TCGASpliceSeq databases. By Lasso regression, five genes were screened to construct the prediction model, with a good reliability by ROC curve. Additionally, results in both univariate and multivariate Cox regression analysis confirmed the well prognosis efficacy of the prediction model (both *P* < 0.001). Moreover, a potential splicing regulatory network was established and after multiple-database validation, we supposed that the signaling axis of HSPB1 up-regulating the PIP5K1C − 46,721 − AT (*P* < 0.001) might mediate the tumorigenesis, progression and metastasis of PRAD via the key members of Alzheimer’s disease pathway (SRC, EGFR, MAPT, APP and PRKCA) (*P* < 0.001).

**Supplementary Information:**

The online version contains supplementary material available at 10.1007/s10528-023-10367-z.

## Introduction

Cancer and other noncommunicable diseases (NCDs) are now widely recognized as a threat to global development. Unfortunately, it lacks a global solution. Many highly effective prevention and treatment strategies exist for cancer. However, they are often very specific (e.g. vaccination for human papillomavirus and hepatitis B virus for prevention of cervical and liver cancer, or tyrosine kinase inhibitors for cancers with targetable mutations). Therefore, most cancers do not have corresponding precise treatment schemes, especially for advanced cancer, such as advanced prostate cancer, and often have distant metastasis, which increases the difficulty of treatment and reduces the survival expectation of patients (Maguire et al. 2015; Martínez-Montiel et al. 2017; Fitzmaurice, et al. 1990). As one of the most general malignancies of urinary system, prostate adenocarcinoma (PRAD) is the fifth chief cause of cancer mortality in men (Siegel et al. 2013; Ferlay et al. 2015). The incidence of prostate cancer seems more likely to be correlated with age, with its highest interval between ages 75–79 (Grozescu and Popa 2017). Distant metastasis often occurs in advanced PRAD (Smith et al. 2018) and in addition to regional lymph nodes, bone was the most common site of metastasis (Coleman 1997; Hayward et al. 1987; Whitmore 1984). In addition, in the late stage of prostate cancer, it appears or shows a phenomenon of treatment resistance, which is called ‘neuroendocrine prostate cancer (NEPC)’, which is an invasive subtype of prostate cancer. Patients with NEPC have frequent visceral or lytic bone metastases, low PSA levels and frequent RB1 and TP53 gene loss. In particular, we found that patients with newly diagnosed NEPC and histologically simple small cell carcinoma have a worse prognosis than patients with mixed NEPC (Conteduca, et al. 2019). At the same time, the site of bone metastasis, such as spine, vertebral body or long bone shaft, is often prone to pathological fracture and even spinal cord compression, which often indicates that the overall survival rate of patients in advanced stage is significantly reduced (Conteduca et al. 2019). Generally, PRAD patients with bone metastases had 1.5 times tendency to die than those with lymph node metastases (Gandaglia et al. 2015).Thus, it is of urgent need to explore the mechanism of distant metastasis, in especial bone metastasis and forecast the prognosis of patients with PRAD.

Alternative splicing (AS) is one of the main engines driving proteome diversity. In addition to being a key mechanism for development, cell regulation and differentiation of cell-type-specific functions (Norris and Calarco 2012), alternative splicing is also in the process of a variety of pathologies (Chabot and Shkreta 2016). It is estimated that more than 94% of genes are selectively splicing in humans and many isoforms are particularly related to cancer progression and metastasis, which means that there may be a relatively small number of splicing factors or their regulators driving multiple oncogenic processes (Oltean and Bates 2014). Alternative splicing (AS) is a marker of cancer and a potential target of new anticancer therapy. It is well-known that breast cancer related as events are related to disease progression, metastasis and survival of breast cancer patients (Oh and Pradella 2021). Nowadays, although the mechanism of alternative splicing had been reported (Oltean and Bates 2014; Climente-Gonzalez et al. 2017), the relationship between AS and tumor progression in PRAD had not been exhaustively described.

In this study, all the PRAD samples information including RNA-seq data, clinical information and splicing factors were saved from The Cancer Genome Atlas (TCGA) database. Meanwhile, alternative splicing event (ASE) data were gained from TCGASpliceSeq database (Ryan et al. 2016). Additionally, univariate and multivariate Cox regression were performed to screen ASEs with prognostic value. A regulatory network of co-expressed splicing factors (SFs) and ASEs was also constructed, along with the co-expression relationship among Kyoto Encyclopedia of Genes and Genomes (KEGG) pathway, to discover ASEs with prognostic value and noteworthy relationship with distant metastasis, especially bone metastasis. Thus, our findings provide prognostic and metastatic molecular biomarkers as well as potential therapeutic targets for PRAD patients.

## Materials and Methods

### Data Collection

RNA-seq data, clinical information and SFs of PRAD samples were obtained from TCGA database (https://tcgadata.nci.nih.gov/tcga/). At the same time, ASEs data were gained from the TCGASpliceSeq database (https://bioinformatics.mdanderson.org/TCGASpliceSeq/) (Ryan et al. 2016) which included seven types (alternate acceptor site, AA; exon skip, ES; alternate terminator, AT; mutually exclusive exons, ME; retained intron, RI; alternate donor site, AD; alternate promoter, AP) (Chen et al. 2019). The dataset included 500 PRAD patients, and each tumor case had matching corresponding entries in the TCGASpliceSeq database. The gene name, the ID number of the TCGASliceSeq database (AS ID) and alternative splicing type were the composition of each ASE ID. Taking the annotation term “SLC9B1-70,159-AT” as an example, the gene name was SLC9B1, the AS ID was 70,159 and the splicing pattern was AT. Baseline information, such as gender, age, TN staging, clinical stage, survival time and survival status were collected.

### Identification of OS-SEs

To identify and analyze OS-SEs, univariate Cox regression analysis was applied. Then, the OS-SEs were presented in seven categories according to the splicing pattern, respectively. In the Gene Ontology (GO) term and KEGG pathway of the genes in OS-SEs, the top 20 enrichments terms were selected to further analyze.

### Construction of the Prognostic Model According to the OS-SEs

On the top 20 OS-SEs that had the highest prognostic values for each type of splicing pattern, Lasso regression and multivariate Cox regression were carried out. Firstly, the Lasso regression was applied to remove the genes with high correlation to avoid an overfitting prognostic model. Then, the regression coefficient of each integrated OS-SE in the prognostic model was evaluated by multivariate Cox regression. Thus, the risk score could calculated according to the following formula:

$${\mathrm{\beta OS}-\mathrm{SE}}_{1}\times {\mathrm{PSIOS}-\mathrm{SE}}_{1}+{\mathrm{\beta OS}-\mathrm{SE}}_{2}\times {\mathrm{PSIOS}-\mathrm{SE}}_{2}+\cdots +{\mathrm{\beta OS}-\mathrm{SE}}_{\mathrm{n}}\times {\mathrm{PSIOS}-\mathrm{SE}}_{\mathrm{n}}$$

in which ${\mathrm{\beta OS}-\mathrm{SE}}_{\mathrm{i}} (\mathrm{i}=1,\cdots ,\mathrm{n})$ represented the coefficient generated by Cox regression and ${\mathrm{PSIOS}-\mathrm{SE}}_{\mathrm{i}} (\mathrm{i}=1,\cdots ,\mathrm{n})$ represented the percent-spliced-in (PSI) for each SE related with survival. Based on the median risk score, all the patients were divided into high-risk group and low-risk group. Then, the efficacy of the prognostic model was evaluated by the area under receiver operator characteristic (ROC) curve. Meanwhile, Kaplan–Meier survival curves were generated to display the significance of difference between survival curves of high-risk group and low-risk group. Finally, univariate and multivariate independent prognostic analysis of OS-associated clinical features and risk score were done to confirm it as an independent prognostic factor.

### Construction of the Interaction and Correlation Network in PRAD

In the study, 390 splicing factors were retrieved from the SpliceAid2 database (Piva et al. 2009). Pearson correlation analysis was applied to identify the interaction and correlation between SFs and OS-SEs. Using Cytoscape (3.7.1) (Shannon et al. 2003), the regulation network of SFs and OS-SEs was generated and the absolute value of cutoff correlation coefficient was 0.450 and the P value was 0.001.

### Recognition of Distant Metastasis and/or Bone Metastasis OS-SEs

Kruskal–Wallis test and Mann–Whitney-Wilcoxon test were carried out to analyze the OS-SEs related to distant metastasis and/or bone metastasis, and they were presented by Beeswarm plots. In addition, these distant metastasis and/or bone metastasis OS-SEs were also displayed in the regulation network.

### Recognition of KEGG Pathways Co-Expressing with ASEs

The univariate Cox analysis was applied to recognize the prognosis-related signaling pathways recognized by Gene Set Variation Analysis (GSVA) (Hanzelmann et al. 2013). Then, distant metastasis and/or bone metastasis and prognosis-related KEGG pathways were brought into the co-expression analysis to explore the potential downstream mechanism of OS-SEs.

### Multidimensional Validation

By examining the expression levels of co-expressed genes and key molecules in all the other sources we found, multiple-database validations, including CCLE (Ghandi 2019), cBioPortal for Cancer Genomics (Gao, et al. 2013), Gene Expression Profiling Interactive Analysis (GEPIA) (Tang et al. 2017), UALCAN (Chandrashekar et al. 2017), LinkedOmics (Vasaikar et al. 2018), PROGgeneV2 (Goswami and Nakshatri 2014), The Human Protein Atlas (Uhlen, et al. 2015) and String (Szklarczyk et al. 2019) were applied to reduce bias.

### Statistical Analysis

For all statistical analyses, only two-sided *P* < 0.05 was regarded as statistically significant. All statistical analyses were achieved by R version 3.5.1 software (Institute for Statistics and Mathematics, Vienna, Austria; www.r-project.org) (Packages: edgeR, ggplot2, rms, glmnet, preprocessCore, survminer, timeROC).

## Results

### Analysis of ASEs and OS-SEs in PRAD

**Fig. 1:**
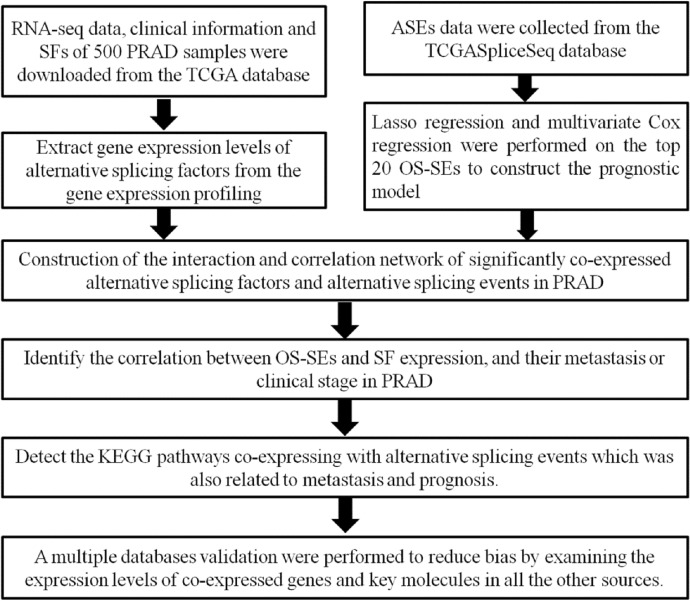
Overall idea design of the study

**Fig. 2:**
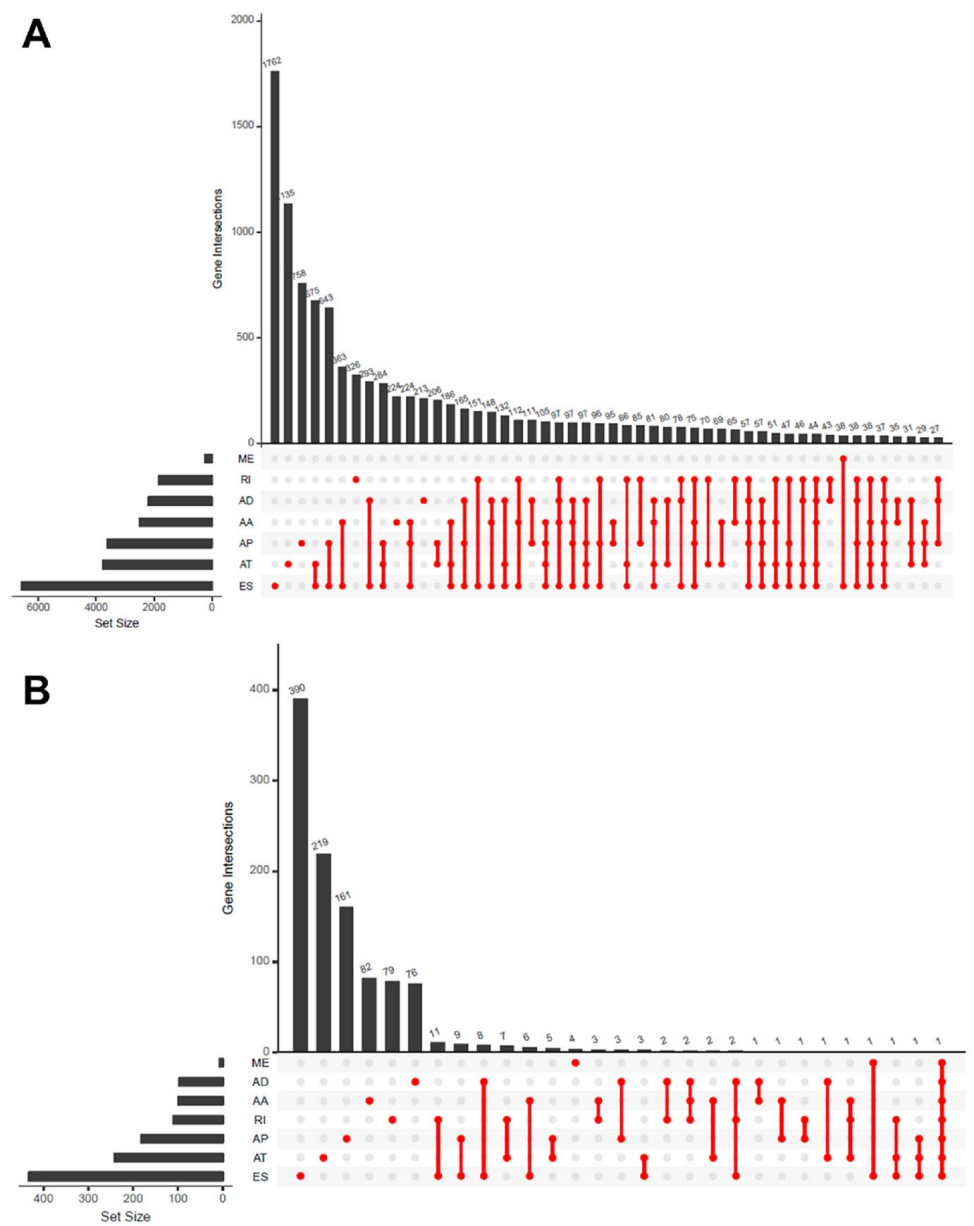
The Upset plot of SEs and OS-SEs. **A** The number of ASEs in different types of splicing patterns; **B** The number of OS-SEs in different types of splicing patterns. *AA* alternate acceptor, *AD* alternate donor, *AP* alternate promoter, *AT* alternate terminator, *ES* exon skip, *ME* mutually exclusive exons, *RI* retained intron

**Fig. 3:**
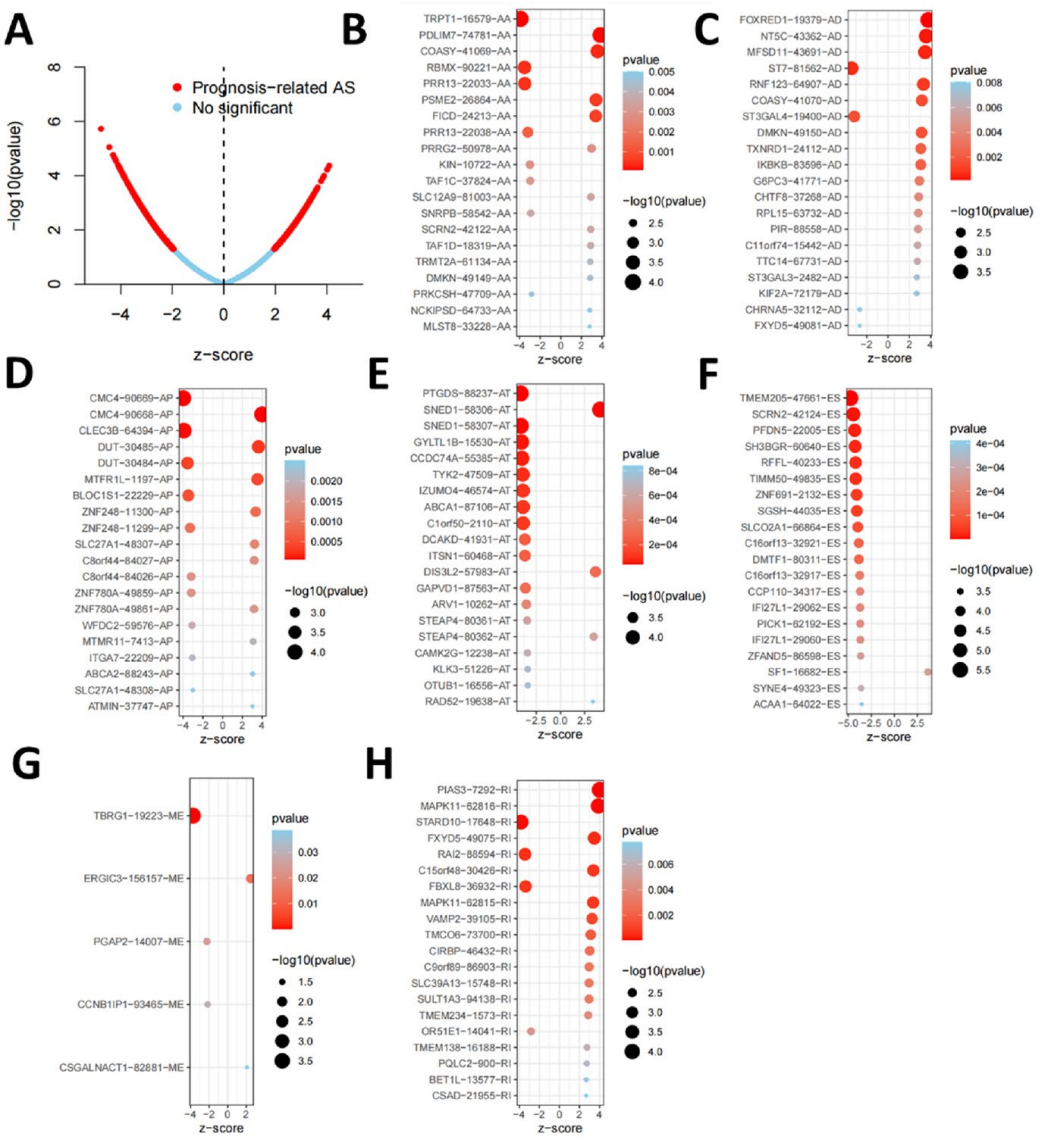
Enrichment analysis of ASEs and seven bubble charts showing the top 20 OS-SEs in seven types of splicing patterns. **A** The volcano plot displaying the prognosis-related and no significant ASEs, respectively. **B** Bubble chart of AA. **C** Bubble chart of AD. **D** Bubble chart of AP. **E** Bubble chart of AT. **F** Bubble chart of ES. **G** Bubble chart of ME. **H** Bubble chart of RI. *AA* alternate acceptor, *AD* alternate donor, *AP* alternate promoter, *AT* alternate terminator, *ES* exon skip, *ME* mutually exclusive exons, *RI* retained intron

The overall analysis procedure is shown in Fig. 1. Table S1 summarized the basic information of 500 patients diagnosed with prostate adenocarcinoma. The ASEs in pooled mRNA samples from 500 PRAD cases collected in the TCGA dataset were analyzed. Each ASE was presented with the gene name, AS ID and splicing type. The number of genes involved in the entire ASEs was illustrated by the Upset plot (Fig. 2A). Entire ASEs in 19,403 genes were recognized in the cohort of PRAD cases: AA events in 2111 genes, AD events in 2193 genes, AP events in 3402 genes, AT events in 3587 genes, ES events in 6403 genes, ME events in 38 genes and RI events in 1669 genes. Meanwhile, a total of OS-SEs in 1167 genes were identified (Fig. 2B). As the Upset plot suggested, ES was the most universal splicing patterns correlated with PRAD prognosis. The volcano plot displayed the distribution of ASEs related or unrelated to overall survival (OS) (Fig. 3A). Seven bubble plots were created to show the top 20 OS-SEs of seven types of splicing patterns, in which the size and color of bubbles represent the value of ASEs for overall survival (Fig. 3B–G).

### Construction of the Prediction Model

**Fig. 4:**
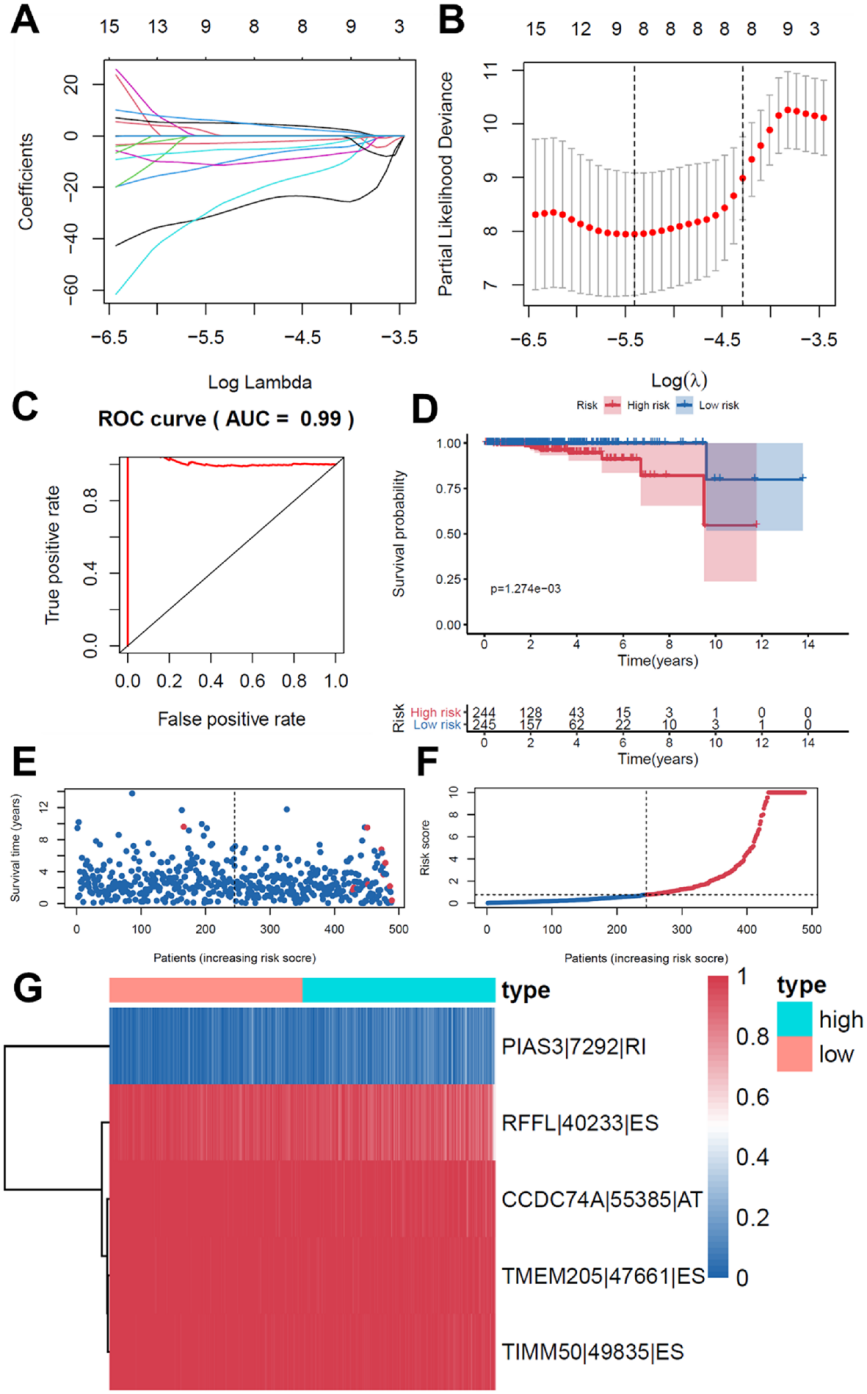
Establishment and assessment of the prediction model. **A**, **B** Lasso regression for OS-SEs removing high correlation genes to prevent over-fitting of the model. **C** The ROC curves demonstrating the accuracy of the model (AUC: 0.99). **D** Kaplan–Meier survival curves for patients in the low and high subgroups of the prediction model demonstrating that risk score could significantly forecast the prognosis of patients with PRAD. **E** The scatter plot showing the trend of change in risk value and the increase in patient mortality as the risk increased and illustrating the clinical status with green and red dots corresponding to survival and death, respectively. **F** The risk curve of each sample ranking by patients’ riskscore from low to high. **G** The heatmap of expression level of five OS-SEs filtered by Lasso regression. *AA* alternate acceptor, *AD* alternate donor, *AP* alternate promoter, *AT* alternate terminator, *ES* exon skip, *ME* mutually exclusive exons, *RI* retained intron

The top 20 OS-SEs with the lowest P value for variable screening were subjected to the Lasso regression. And the genes with the lowest cross-validation error were incorporated as independent prognostic markers of PRAD patients to the final Cox regression model (Fig. 4A, B). After that, each patient's risk score was computed. All patients were divided into two groups based on their median risk score: high-risk and low-risk. Following that, the Kaplan–Meier approach demonstrated that the risk score prediction model between the low-risk and high-risk groups had a strong efficacy (*P* = 0.001). (Fig. 4D). In addition, ROC curves were generated, and the area under the ROC curve (AUC = 0.99) demonstrated the models' high predictive efficiency (Fig. 4C). Because just a handful of the 500 individuals with PRAD died, the AUC may be inaccurate. Furthermore, a risk curve was created, ranking patient risk from low to high, to show that the high-risk group will have a higher death rate (Fig. 4E, F). PIAS3-7292-RI was identified as a high-risk ASE, whereas RFFL-40233-ES, CCDC74A-55385-AT, TMEM205-47,661-ES, and TIMM50-49,835-ES were identified as low-risk ASEs. And these five ASEs made up the prediction model (Fig. 4G).

### Independent Prognostic Analysis

**Fig. 5:**
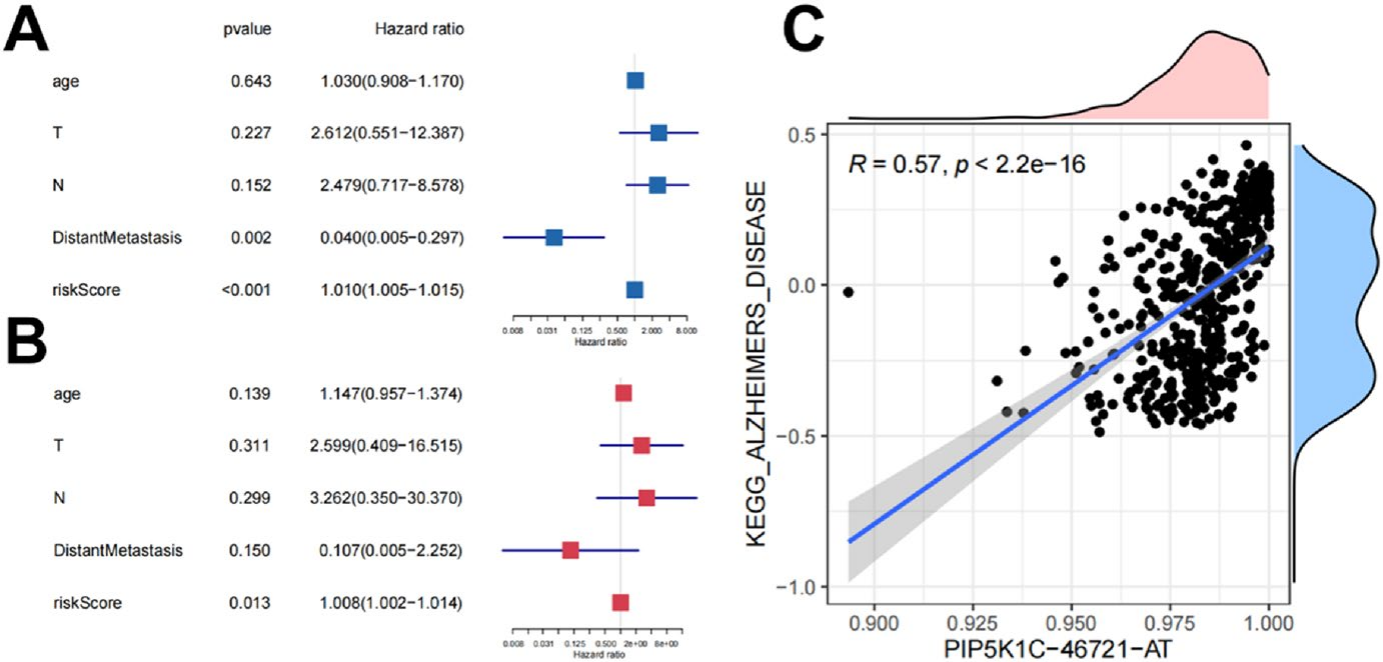
Cox regression analysis for assessing the independent prognostic value of the risk score and corHeatmap of KEGG pathways. **A** univariate and (**B**) multivariate Cox regression analysis verify that risk score can be the independent prognostic factor of BLCA. **C** It shows that PIP5K1C-46721-AT is highly positively correlated with KEGG pathway of Alzheimer's disease

To see if the built prognostic model was independent of other clinical characteristics including age, gender, and tumor stage, researchers used univariate and multivariate Cox regression analysis. Both univariate (hazard ratio (HR), 95 percent confidence interval): 1.010 (1.005–1.015), *P* 0.001) and multivariate (HR, 95 percent confidence interval: 1.008 (1.002–1.014), *P* = 0.013) Cox regression analysis confirmed that risk score could be considered an independent prognostic factor and that the constructed prediction model had good prognosis efficacy (Fig. 5A, B).At the same time, the maps of shear factors and related molecular pathways are used to show the correlation between them(Fig. 5C).

### Correlation Among OS-SEs and SF Expression, and their Metastasis or Clinical Stage

**Fig. 6:**
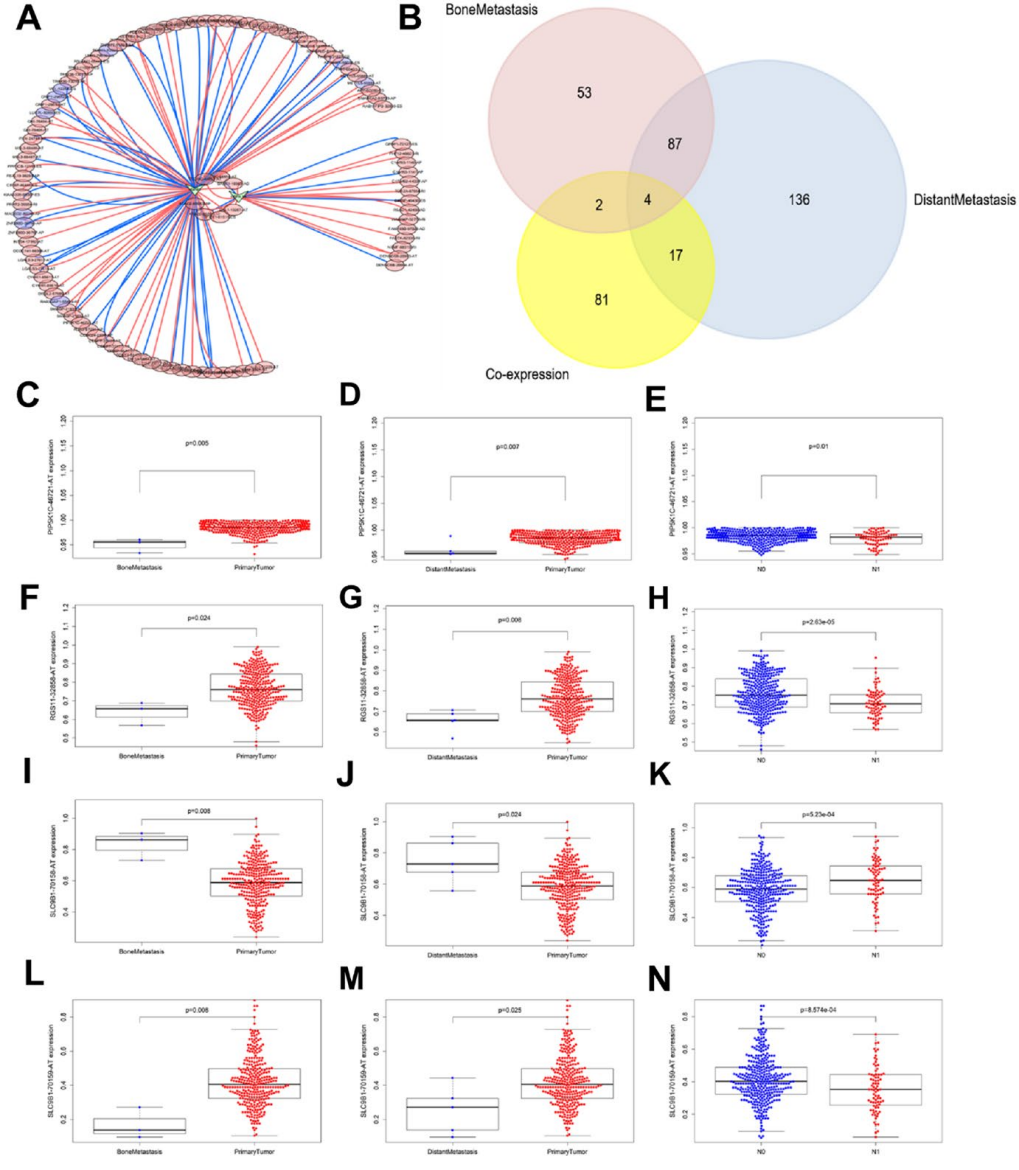
Alternative splicing network and clinical relevance. **A** Regulatory network of notably co-expressed alternative splicing factors and alternative splicing events. The shape of arrow represents the splicing factor, the red circle shows high-risk alternative splicing and the purple circle shows low-risk alternative splicing. The red and blue lines represent the positive and negative regulatory relationships between AS and SF, respectively. **B** Venn plot OS-SEs related to regional lymph node, distant metastasis and bone metastasis. **C** Beeswarm plots displaying PIP5K1C-46721-AT significantly related to bone metastasis. **D** Beeswarm plots displaying PIP5K1C-46721-AT significantly related to distant metastasis. **E** Beeswarm plots displaying PIP5K1C-46721-AT significantly related to regional lymph node. **F** Beeswarm plots displaying RGS11-32,858-AT significantly related to bone metastasis. **G** Beeswarm plots displaying RGS11-32,858-AT significantly related to distant metastasis. **H** Beeswarm plots displaying RGS11-32,858-AT significantly related to regional lymph node. **I** Beeswarm plots displaying SLC9B1-70,158-AT significantly related to bone metastasis. **J** Beeswarm plots displaying SLC9B1-70,158-AT significantly related to distant metastasis. **K** Beeswarm plots displaying SLC9B1-70,158-AT significantly related to regional lymph node. **L** Beeswarm plots displaying SLC9B1-70,159-AT significantly related to bone metastasis. **M** Beeswarm plots displaying SLC9B1-70,159-AT significantly related to distant metastasis. **N** Beeswarm plots displaying SLC9B1-70,159-AT significantly related to regional lymph node

Figure 6A illustrated the possible splicing regulatory network of SFs and OS-SEs. Among them, HSPB1 was associated with 41 favorable OS-SEs (purple ellipses) negatively (blue lines) and 49 adverse OS-SEs (red ellipses) positively (red lines); HSPA1A was associated with 6 favorable OS-SEs (purple ellipses) negatively (blue lines) and 15 adverse OS-SEs (red ellipses) positively (red lines). Then, PIP5K1C-46721-AT, RGS11-32,858-AT, SLC9B1-70,158-AT and SLC9B1-70,159-AT were significantly related to distant metastasis, bone metastasis and clinical stage in the Venn plot (Fig. 6B). Besides, the 4 OS-SEs were presented by Beeswarm plots (Fig. 6C–N).

### Functional Enrichment Analysis

**Fig. 7:**
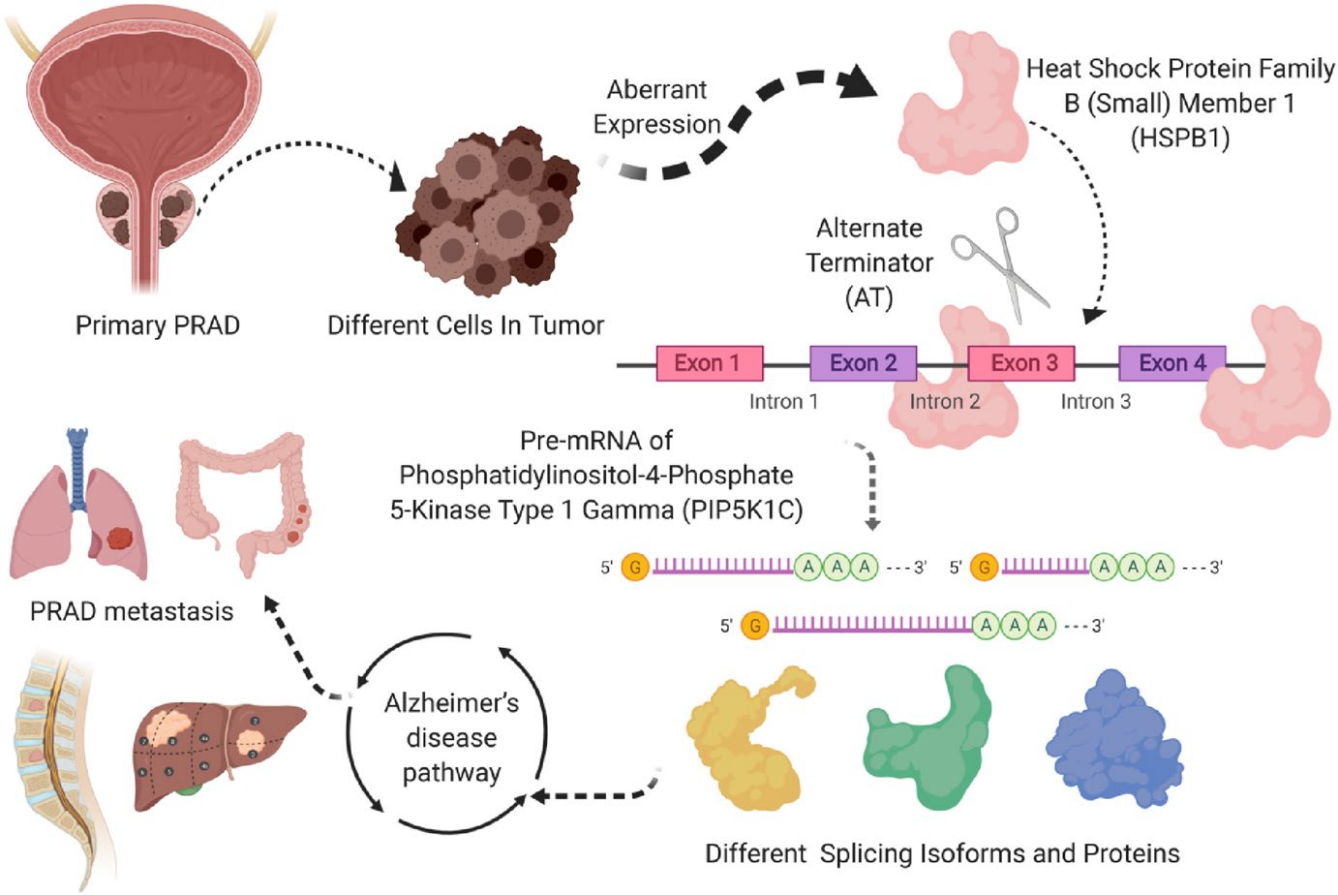
The whole mechanism of HSPB1 regulating the PIP5K1C-46721-AT and playing an essential role in bone metastasis and distant metastasis of PRAD through the Alzheimer’s disease pathway

By GSVA analysis, we found that a total of 185 KEGG pathways were related to the OS (Figure S9). All of them were put into the Pearson correlation analysis with PIP5K1C-46721-AT, RGS11-32,858-AT, SLC9B1-70,158-AT and SLC9B1-70,159-AT to illustrate their co-expression patterns. According to the results, PIP5K1C-46721-AT was extremely co-expressed with the pathway of Alzheimer’s disease pathway (R = 0.480, *P* < 0.001); RGS11-32,858-AT was up-regulated in amyotrophic lateral sclerosis pathway (R = 0.470, *P* < 0.001); SLC9B1-70,158-AT was less expressed in pathway of oxidative phosphorylation pathway (R = − 0.440, *P* < 0.001); SLC9B1-70,159-AT was significantly correlated with oxidative phosphorylation pathway (R = 0.440, *P* < 0.001). By multidimensional validation, we wondered that HSPB1 regulating the PIP5K1C-46721-AT might play an essential part in bone metastasis and distant metastasis of prostate adenocarcinoma through the Alzheimer’s disease pathway, which was also related to prognosis. And the whole mechanism of action was displayed in Fig. 7.

### Multidimensional Validation

In the pathway unification database, SRC, EGFR, MAPT, APP and PRKCA were identified as key molecules in the pathway of Alzheimer’s disease. Next, the 7 OS-SEs were verified by multiple databases (Table S2). In the database of CCLE, 6 OS-SEs except for MAPT were high-expressed in tissue level in PRAD (Figure S1). In cBioPortal for Cancer Genomics database, HSPB1, APP and PRKCA were high-expressed while PIP5K1C, EGFR, MAPT were low-expressed in tissue level in PRAD (Figure S2). And the results of external validation using GEPIA suggested that HSPB1 PIP5K1C and PRKCA expressed highly in normal tissue and lowly in PRAD, APP expressed lowly in normal thyroid and highly in PRAD (Figure S3). Then, in UALCAN database, HSPB1, PIP5K1C, EGFR, APP and PRKCA were high-expressed and were related to expression significantly (Figure S4). Meanwhile, PRKCA was also associated with OS significantly in LinkedOmics database (Figure S5). The results of the human protein atlas suggested that SRC were higher expressed, on the other hand, APP and PRKCA were lower expressed in the protein levels (Figure S7). Furthermore, the correlation of the 7 OS-SEs was detected in the String database (Figure S8).

## Discussion

Globally, the risk of prostate cancer is 1 in 18. Since 2007, the incidence rate has been increasing. With the aging and increasing population, the incidence of prostate cancer has increased by 42% (940 thousand in 2007 and 1 million 300 thousand in 2017). 21% of this increase can be attributed to changes in age structure of the population, 13% attributable to changes in population size, and 8% attributable to changes in age specific incidence rate. Prostate cancer was the highest incidence rate of cancer in 114 countries in 2017 and the leading cause of cancer-related deaths in 56 countries (Fitzmaurice et al. 1990). Prostate adenocarcinoma is the second most challenging cause of cancer-related deaths in men (Siegel et al. 2013; Siegel et al. 2014) and metastasis is the main cause for its high mortality (Siegel et al. 2014). Though androgen withdrawal is considered as the mainstay of treatment for metastatic PRAD patients, death is likely to occur due to androgen-independent progression shortly after treatment (So et al. 2007). Alternative splicing is an important mechanism for proteome diversity (Liu et al. 2017) and also involved in tumor progression and metastasis (Oltean and Bates 2014). Hence, there is a substantial need to identify the function of OS-SEs, SFs and signaling pathways in the metastasis, tumorigenesis and prognosis of PRAD patients. In this study, a prediction model with well prognosis efficacy was constructed and a potential splicing regulatory network was set upto demonstrate the relationship among OS-SEs, SF expression and their metastasis. Moreover, by multiple databases, we speculated that for PRAD patients with metastasis, HSPB1 could up-regulating the PIP5K1C − 46,721 − AT by the pathway of Alzheimer’s disease which was also related to prognosis.

Heat shock protein 27 (HSPB1, HSP27), an ATP independent small molecular chaperone, is highly expressed in aggressive cancers and plays an indispensable role in protein homeostasis, transport processes and signal transduction (Vahid et al. 2016). In this study, HSPB1 was one of the SFs, which linked OS-SEs were related to OS and metastasis in the potential splicing regulatory network. Similar to our results, previous studies had shown that HSPB1 acted as a biomarker for survival prediction in late metastatic prostate cancer (Cho et al. 2018). It can increase the invasion of PRAD cells, and the over-expression of HSPB1 could increase primary tumor mass and metastases (Voll et al. 2014; Vasiljevic et al. 2013). Additionally, HSPB1-induced prostate cancer cell motility and metastatic progression. HSPB1 stimulated the activity of matrix metalloproteinase 2 (MMP-2) through the TGF-β pathway, driving the migration of cancer cells from the prostate gland which is the prerequisite of distant metastasis (Voll et al. 2014; Xu et al. 2006).

Phosphatidylinositol-4-phosphate 5-kinase type-1C (PIP5K1C)is a lipid kinase which adjusts adhesion dynamics and cell attachment via the specificity of phosphatidylinositol-4, 5-diphosphate (PI4,5P_2_) site (Durand et al. 2018). The results of our study demonstrated that aberrant ASE of PIP5K1C was significantly related to poor prognosis and tumor metastasis in PRAD patients. Meanwhile, previous studies revealed that PIP5K1C played a key role in cell migration, invasion and metastasis (Chen et al. 2019). Its depletion inhibited cell migration in HeLa cervical cancer cells and breast cancer cells (Sun et al. 2010; Sun et al. 2007). Furthermore, knockout of PIP5K1C in 4T1 breast cancer cells showed a noteworthy reduction in tumor progression and metastasis (Chen et al. 2015).

Furthermore, a protein-to-protein network was generated by the String database to demonstrate the correlation of SF, ASE and key genes in pathway. And the results confirmed that there was a close connection among HSPB1, PIP5K1C and five key genes in pathway of Alzheimer’s disease (AD) (Alzheimer's 2016). It is reported that PRAD had a high co-occurrence probability with AD in elderly people (Raji et al. 2008). As we know, androgen deprivation therapy (ADT) is the foundation of treatment for PRAD patients (Bagcchi 2016). It is suggested that treatment by ADT for prostate cancer patients could raise the risk of Alzheimer’s disease (Bagcchi 2016). Prostate cancer cells show excessive activation of androgen signaling pathway, resulting in uncontrolled proliferation of tumor cells. The preliminary discovery that hormone regulates the size and function of prostate and the observation that the growth of prostate cancer is affected by androgen provide a basis for androgen deprivation therapy (ADT). In healthy men, androgen testosterone (T) and its derivative dihydrotestosterone (DHT) are essential for cell survival and prostate function. Androgen deprivation therapy (ADT), which aims to reduce testosterone levels, is important for the health and regeneration of neurons. However, the adverse reactions caused by long-term inhibition of testosterone have a great impact on the quality of life of patients (Barata et al. 2019; Crawford et al. 2019). Also, ADT could increase the risk of cardiovascular disease (Nead et al. 2016). These findings may increase the brain’s susceptibility to Alzheimer’s disease. Another evidence showed there was an association between ADT and impairments in visuomotor and executive functioning, which are chief features of Alzheimer’s disease (McGinty et al. 2014; Nelson et al. 2008). Besides, a population-based study supported that relationship between AD and prostate cancer (Lin et al. 2018), which seemed to be explainable in some plausible biologic mechanisms, abnormal neuronal cell death (Raji et al. 2008; Nead et al. 2017; Graham et al. 2017) and deposits of proteins (Ballard et al. 2011; Scheltens et al. 2016).

Nevertheless, this study still had some limitations. Firstly, the sequencing data relied on the only one cohort and the sample size was restricted. Secondly, all the subjects involved in this study were from European, which might cause a selection bias. Thirdly, though all the results were confirmed by external databases, further experiments in vitro and in vivo were still needed. Despite these limitations, we proposed that aberrant HSPB1 regulated PIP5K1C-46721-AT might be linked to the tumorigenesis, metastasis and poor prognosis of PRAD through Alzheimer's disease pathway according to varies of bioinformatics analysis. Meanwhile, our findings offer a good guidance for clinicians in the treatment of PRAD patients with metastasis and prognosis.

In the present study, we proposed that up-expressed HSPB1 positive regulated PIP5K1C-46721-AT might be linked to the tumorigenesis, metastasis and poor prognosis of PRAD through pathways related to Alzheimer's disease. Additionally, a prediction model consisted of PIAS3-7292-RI, RFFL-40233-ES, CCDC74A-55385-AT, TMEM205-47,661-ES and TIMM50-49,835-ES was a successful forecast model for prognosis of PRAD patients, which had a high prediction accuracy. Moreover, we found four alternative splicing events-related to metastasis, they might be potential therapeutic targets for PRAD metastasis.

Personalization of PCa therapy is achievable in the twenty-first century, in addition to identifying individuals who benefit from ADT. Personalized therapy tactics will enhance results when more treatment choices become accessible and more clinically relevant tumor/genetic markers are uncovered. To confirm that testosterone (T) levels of less than 20 ng/dl enhance clinical outcomes, prospective studies can be done. The findings that individuals with metastatic prostate cancer had a greater prevalence of germline mutations in DNA repair genes such as BRCA2, ATM, CHEK2, BRCA1, rad51d, and PALB2 than patients with local illnesses is a promising study field for discovering disease risk levels in gene testing (Pritchard et al. 2016).Future study will discover how gene mutations alter and customize therapy choices, as well as describe the clinical importance of these gene mutations. Similarly, PDL-1 inhibitors have a profound effect on certain individuals with mismatched mutations (Graff et al. 2016). Aside from these two instances, there is a lot of room for novel medication combinations to be discovered and drug indications to be expanded. Patients will profit from these new scientific advancements if personalized medicines based on patient-specific characteristics are developed.

### Supplementary Information

Below is the link to the electronic supplementary material.

Supplementary file1 (DOCX 6211 kb)

## Data Availability

The datasets generated and/or analyzed during the current study are available in the Supplementary Material and TCGA-PRAD program (https://portal.gdc.cancer.gov).

## Abbreviations

AA: Alternate acceptor
AD: Alternate donor
ADT: Androgen deprivation therapy
AP: Alternate promoter
AS: Alternative splicing
ASEs: Alternative splicing events
AT: Alternate terminator
AUC: Area under the ROC curve
ES: Exon skip
GEPIA: Gene Expression Profiling Interactive Analysis
GO: Gene Ontology
GSVA: Gene Set Variation Analysis
HSPB1: Heat shock protein 27
KEGG: Kyoto Encyclopedia of Genes and Genomes
ME: Mutually exclusive exons
MMP-2: Matrix metalloproteinase 2
OS: Overall survival
PIP5K1C: Phosphatidylinositol-4-phosphate 5-kinase type-1C
PI4,5P_2_: Phosphatidylinositol-4, 5-diphosphate; AD, Alzheimer’s disease
PRAD: Prostate adenocarcinoma
PSI: Percent-spliced-in
RI: Retained intron
ROC: Receiver operating characteristic curve
SF: Splicing factor
TCGA: The Cancer Genome Atlas
