# Supplementary material for: Identification of Bone Metastatic and Prognostic Alternative Splicing Signatures in Prostate Adenocarcinoma
Source: Biochem Genet. 2023 Apr 3;61(6):2242–59. doi: 10.1007/s10528-023-10367-z (PMC10665256; doi:10.1007/s10528-023-10367-z)
Supplement: Supplementary file 1 — Supplementary file1 (DOCX 6211 kb) [file 10528_2023_10367_MOESM1_ESM.docx]

**
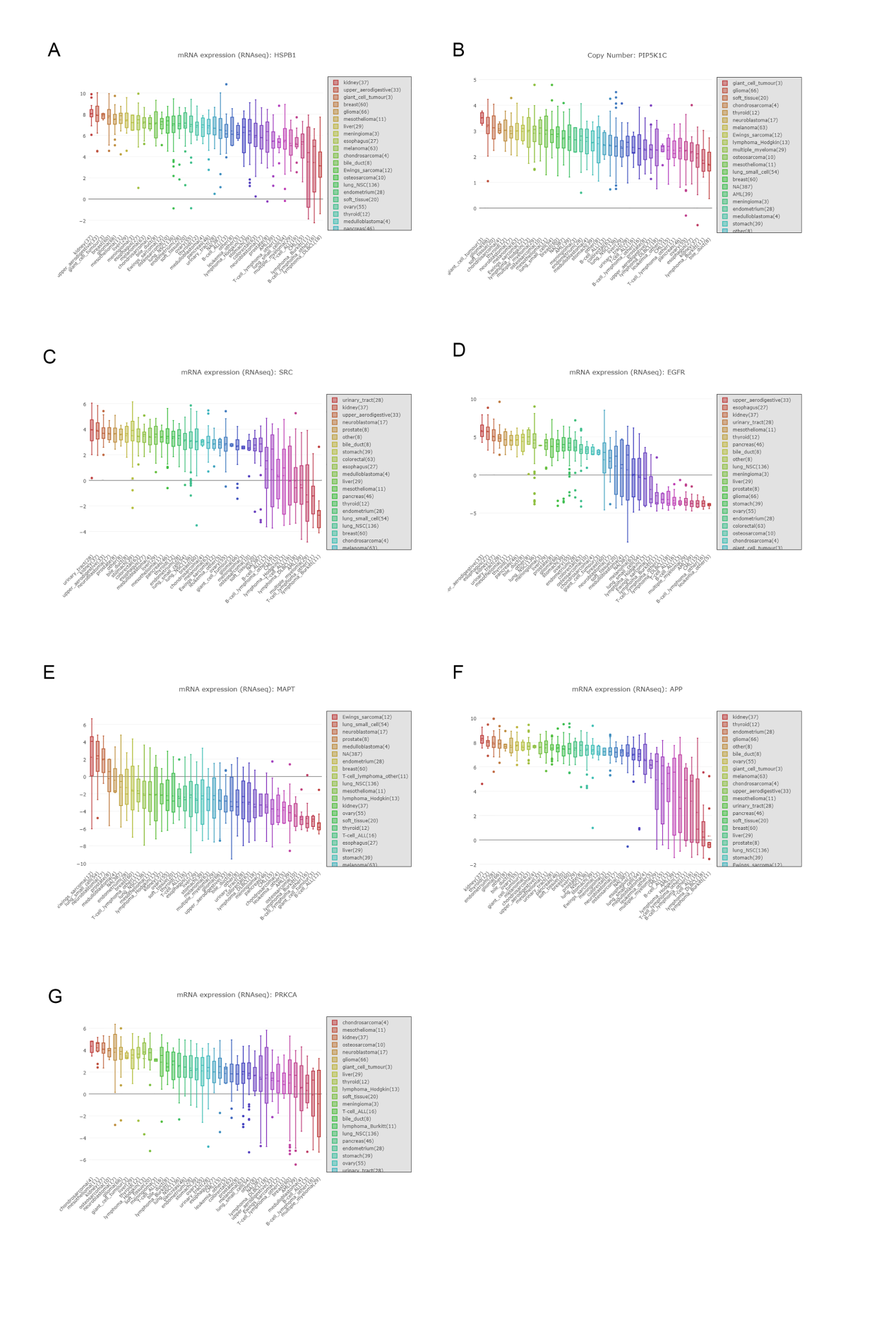
**

**Figure S1** The results of external validation using CCLE.

In CCLE, HSPB1 (A), PIP5K1C (B), SRC (C), EGFR (D), APP (F) and PRKCA (G) were high-expressed while MAPT (E) was low-expressed in tissue level in PRAD.

**
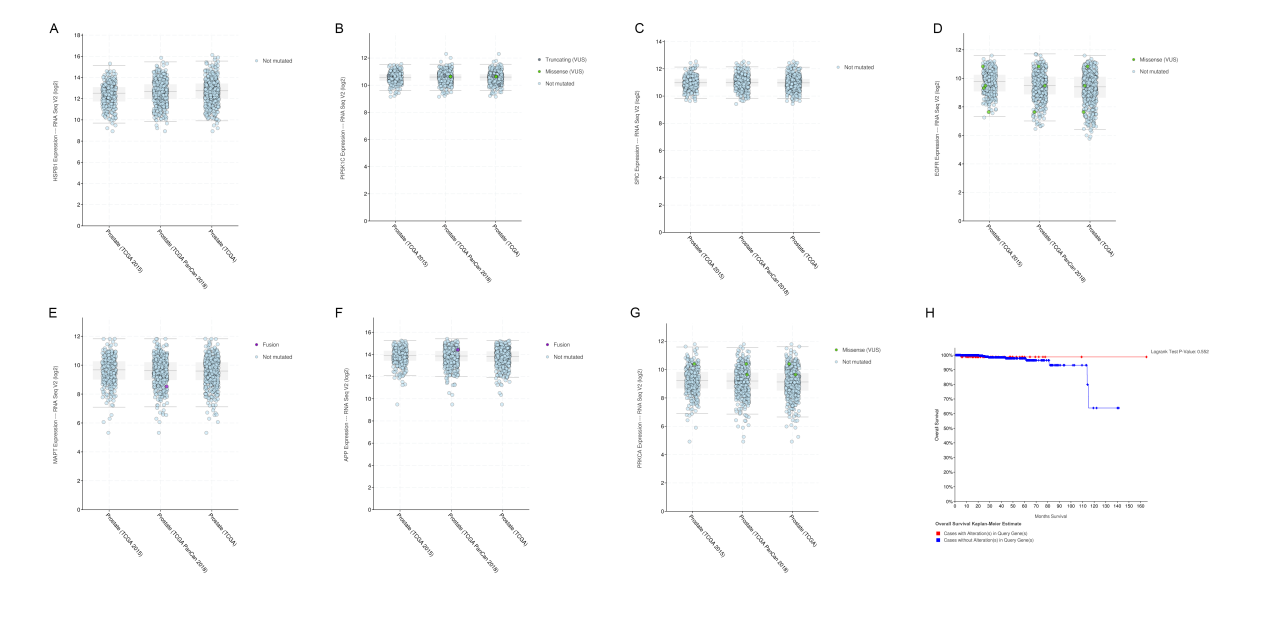
**

**Figure S2** The results of external validation using cBioPortal for Cancer Genomics.

HSPB1 (A), APP (F) and PRKCA (G) were high-expressed while PIP5K1C (B), EGFR (D), MAPT (E) were low-expressed in tissue level in PRAD.

**
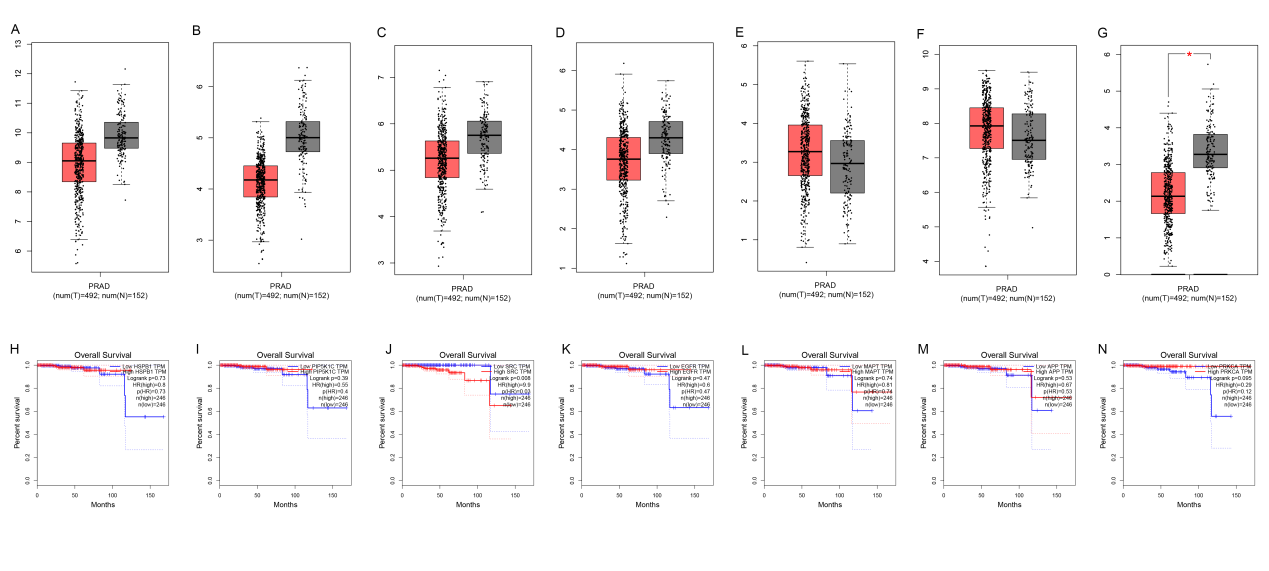
**

**Figure S3** The results of external validation using Gene Expression Profiling Interactive Analysis. HSPB1 (A, H), PIP5K1C (B, I) and PRKCA (G, N) expressed highly in normal tissue and lowly in PRAD; APP (F, M) expressed lowly in normal thyroid and highly in PRAD.(In the box diagram, red represents PRAD and gray represents normal tissue or normal thyroid).

**
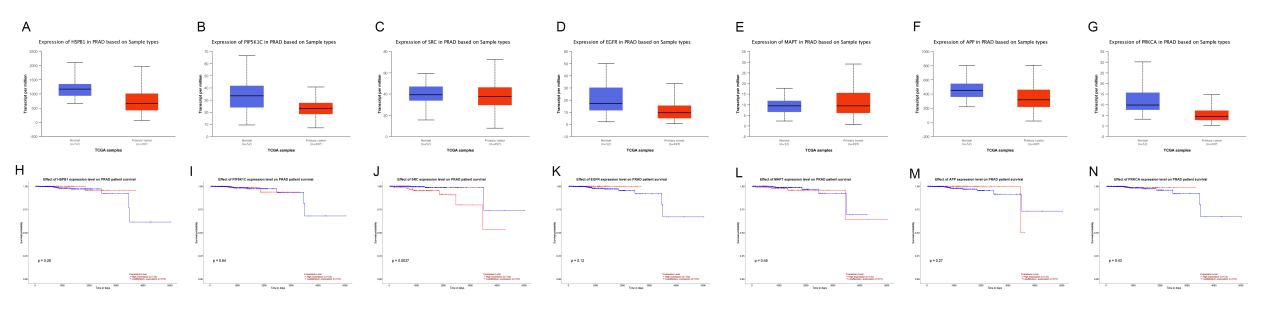
**

**Figure S4** The results of external validation using UALCAN.

HSPB1 (A, H), PIP5K1C (B, I), EGFR (D, K), APP (F, M) and PRKCA (G, N) expressed highly in normal tissue and lowly in PRAD and they were related to expression significantly.

**
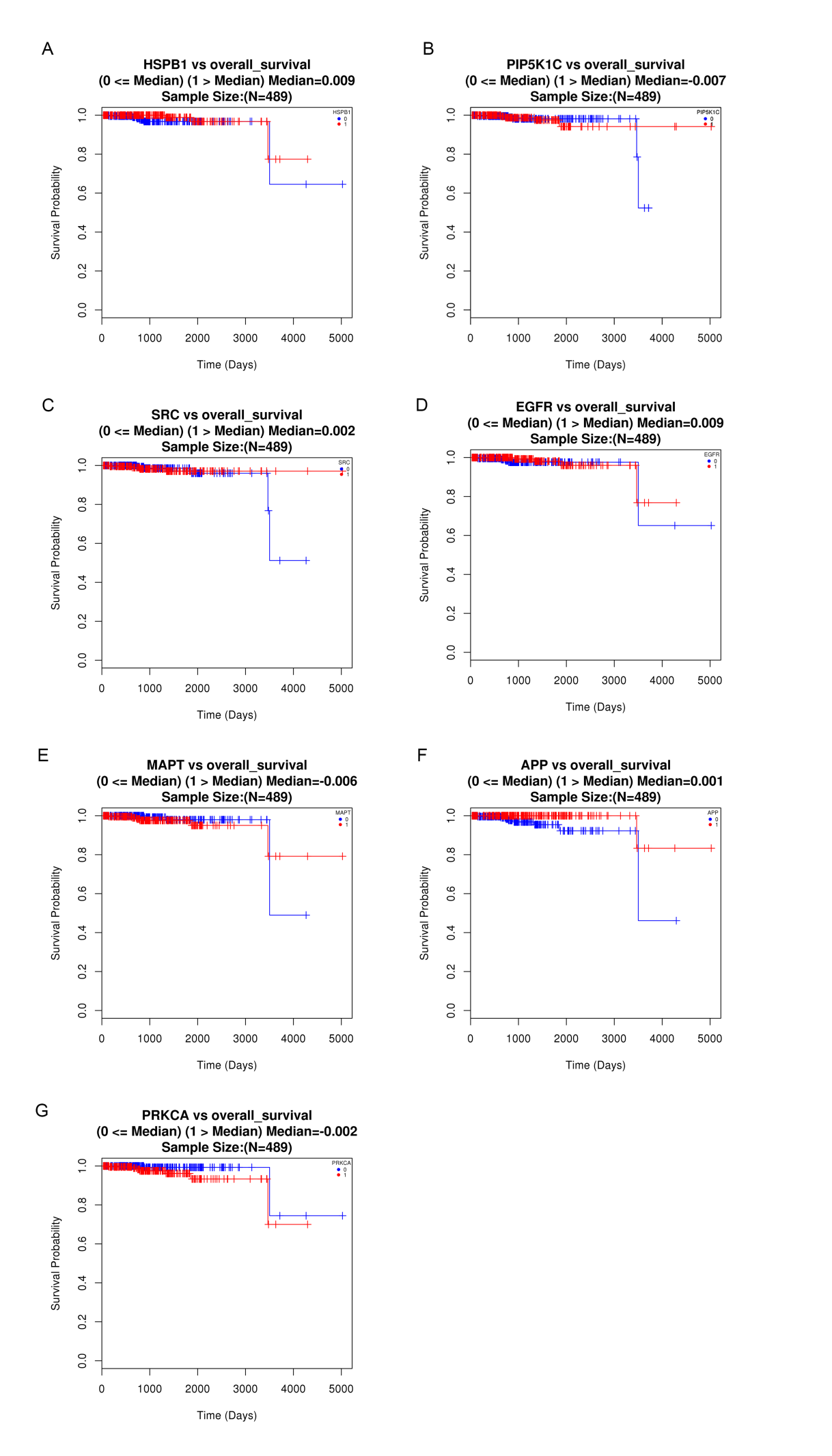
**

**Figure S5** The results of external validation using LinkedOmics.

PRKCA (G) was also associated with OS significantly in LinkedOmics database

**
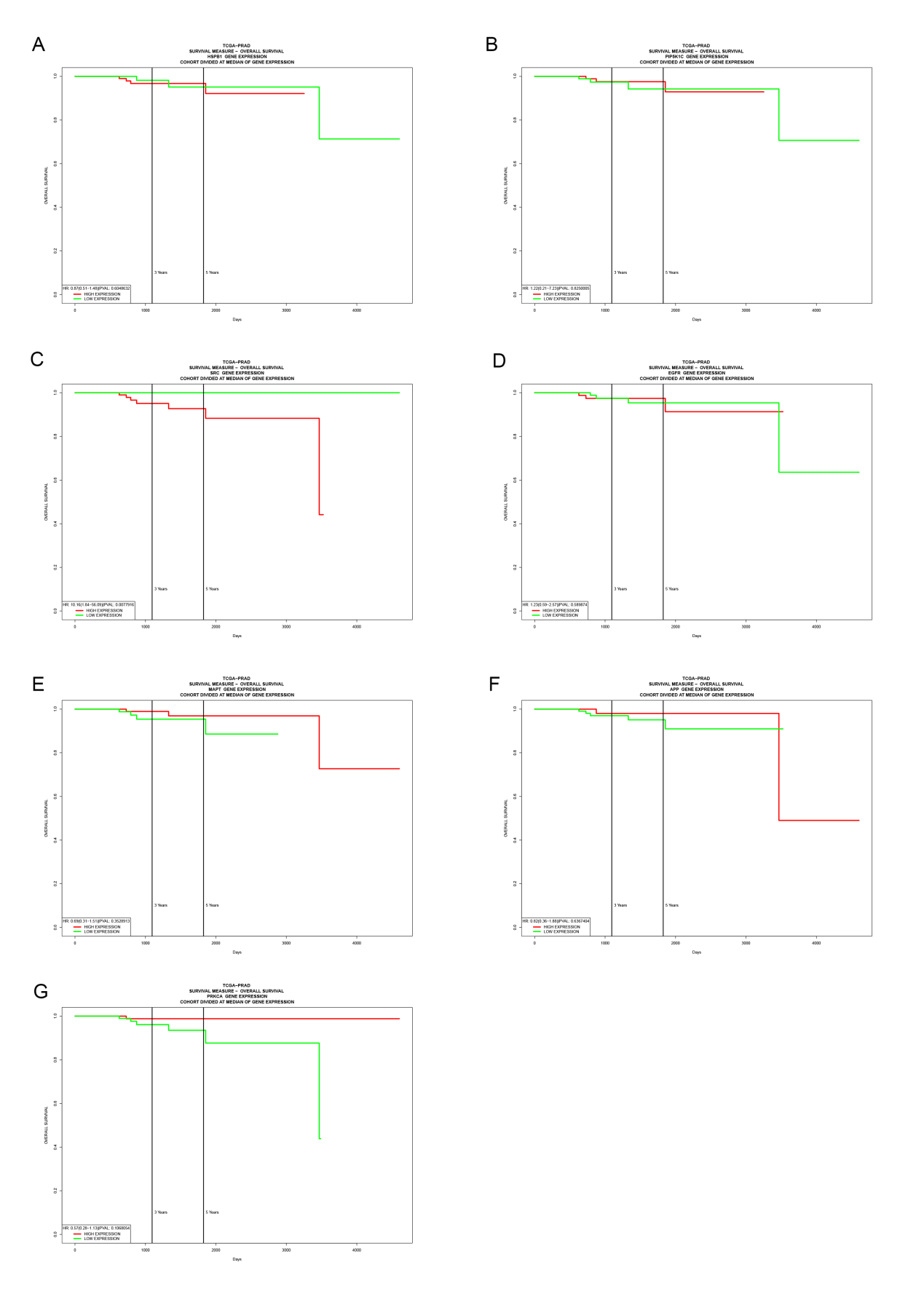
**

**Figure S6** The results of external validation using PROGgeneV2.

**
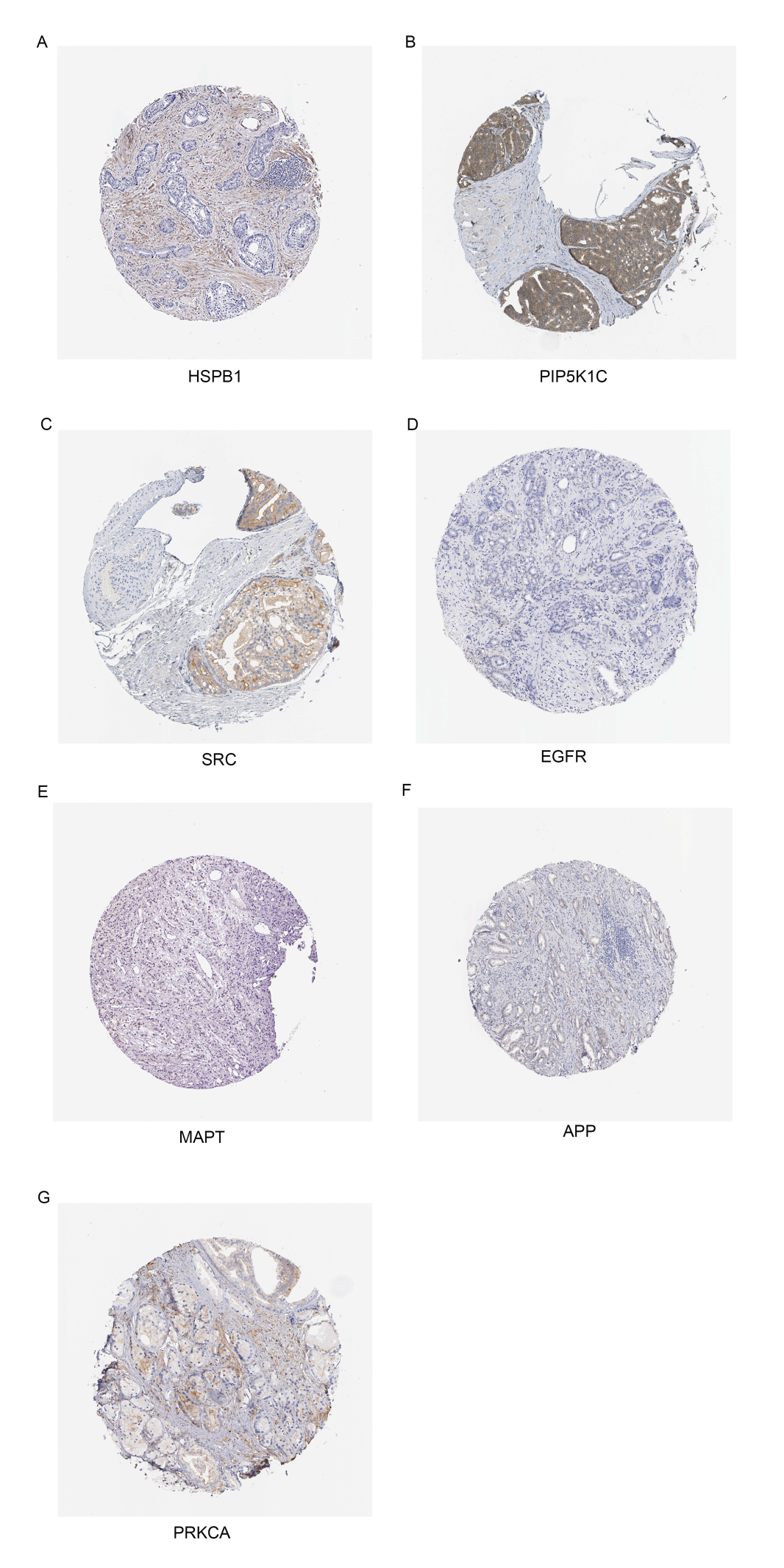
**

**Figure S7** The results of external validation using the human protein atlas.

The results of the human protein atlas suggested that SRC (C) were higher expressed while APP (F) and PRKCA (G) were lower expressed in the protein levels

**
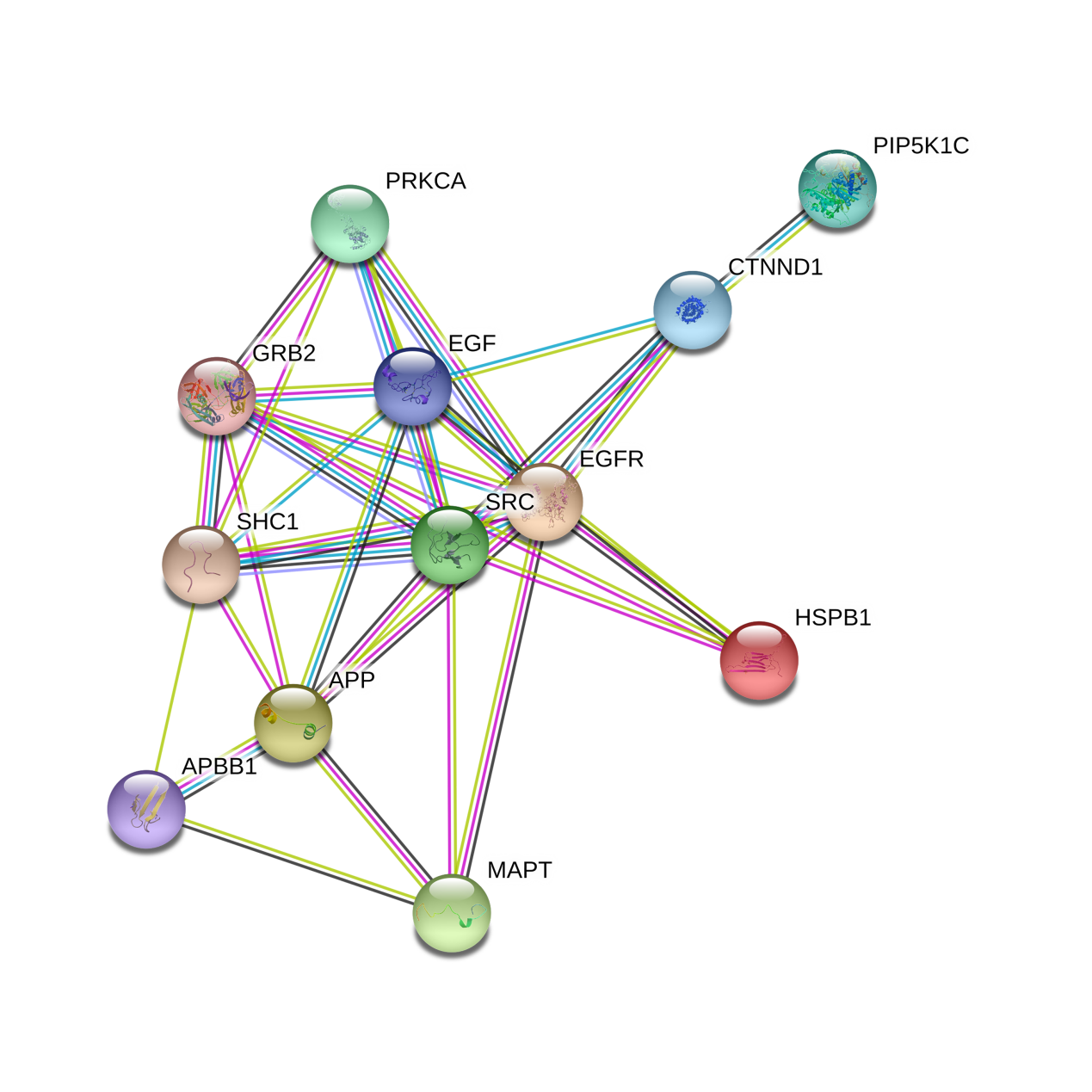
**

**Figure S8** The results external validation using String database confirmed that there was a close connection among the 7 OS-SEs.

**
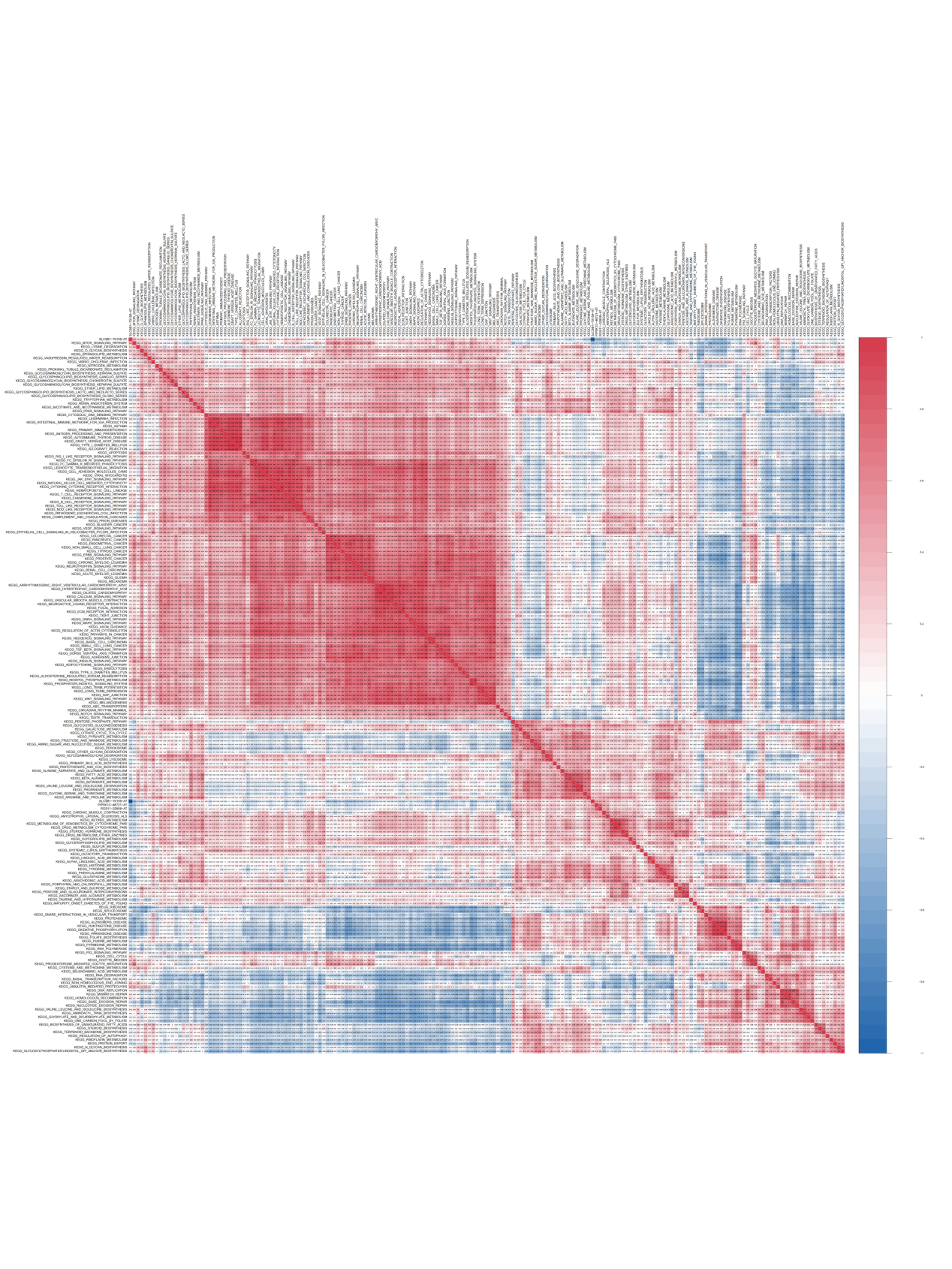
**

**Figure S9** CorHeatmap of KEGG pathways and alternative splicing events that had prognostic value and were significantly associated with bone metastasis. GSVA pathway analysis and univariate Cox regression analysis identified survival related KEGG pathways, and co-expressed alternative splicing events related to prognosis and bone metastasis with survival related KEGG pathways.

| **Table S1** Baseline information of 500 patients diagnosed with prostate adenocarcinoma. | |
| --- | --- |
| Variables | Total Patients (N = 500) |
| **Age, years** |  |
| Mean ± SD | 61.01 ± 6.82 |
| Median (Range) | 61 (41- 78) |
| **Gender** |  |
| Female | 0 (0.00%) |
| Male | 500 (100.00%) |
| **T** |  |
| T2a | 13 (2.60%) |
| T2b | 10 (2.00%) |
| T2c | 165 (33.00%) |
| T3a | 159 (31.80%) |
| T3b | 136 (27.20%) |
| T4 | 10 (2.00%) |
| unknow | 7 (1.40%) |
| **N** |  |
| N0 | 348 (69.60%) |
| N1 | 79 (15.80%) |
| unknow | 73 (14.60%) |
| **Distant Metastasis** |  |
| Distant Metastasis | 5 (1.00%) |
| Primary Tumor | 315 (63.00%) |
| Not available | 180 (36.00%) |
| **Bone Metastasis** |  |
| Bone Metastasis | 3 (0.60%) |
| Primary Tumor | 317 (63.40%) |
| Not available | 180 (36.00%) |
| **Abbreviations:**SD,Standard deviation; T,tumor; M,metastasis; N,regional lymph node. | |

**Table S2** The external validation results of HSPB1, PIP5K1C, SRC, EGFR, MAPT, APP and PRKCA.

| **Database** | **HSPB1**  **(splicing factor gene)** | **PIP5K1C**  **(alternative splicing events gene)** | **SRC**  **(pathway gene)** | **EGFR**  **(pathway gene)** | **MAPT**  **(pathway gene)** | **APP**  **(pathway gene)** | **PRKCA**  **(pathway gene)** |
| --- | --- | --- | --- | --- | --- | --- | --- |
| **CCLE** | Tumor high | Tumor high | Tumor high | Tumor high | Tumor medium | Tumor high | Tumor high |
| **cBioPortal** | Tumor high | Tumor low | No significant difference | Tumor low | Tumor low | Tumor high | Tumor high |
| **GEPIA** | Tumor low  Normal high  K-M: P = 0.730 | Tumor low  Normal high  K-M: P = 0.400 | No significant difference  K-M: P = 0.030 | No significant difference  K-M: P = 0.470 | No significant difference  K-M: P = 0.740 | Tumor high  Normal low  K-M: P = 0.530 | Tumor low  Normal high  K-M: P = 0.120 |
| **UALCAN** | Tumor low  Normal high  Expression: P < 0.001  K-M: P = 0.280 | Tumor low  Normal high  Expression: P < 0.001  K-M: P = 0.940 | No significant difference  Expression: P = 0.598  K-M: P = 0.004 | Tumor low  Normal high  Expression: P < 0.001  K-M: P = 0.120 | No significant difference  Expression: P = 0.004  K-M: P = 0.450 | Tumor low  Normal high  Expression: P < 0.001  K-M: P = 0.270 | Tumor low  Normal high  Expression: P < 0.001  K-M: P = 0.430 |
| **Linkedomics** | K-M: P = 0.001 | K-M: P = 0.739 | K-M: P = 0.002 | K-M: P = 0.285 | K-M: P = 0.438 | K-M: P = 0.010 | K-M: P < 0.001 |
| **PROGgeneV2** | K-M: P = 0.605 | K-M: P = 0.825 | K-M: P = 0.008 | K-M: P = 0.590 | K-M: P = 0.353 | K-M: P = 0.637 | K-M: P = 0.107 |
| **The human protein atlas** | Normal medium | Normal medium | Normal high | Not detected | Not detected | Normal low | Normal low |
